# Impact of Age at Diagnosis on Clinicopathological Features, Prognosis, and Management of Gastric Cancer: A Retrospective Single-Center Experience from Spain

**DOI:** 10.3390/cancers15174241

**Published:** 2023-08-24

**Authors:** Cristina Díaz del Arco, Luis Ortega Medina, Lourdes Estrada Muñoz, Elena Molina Roldán, Soledad García Gómez de las Heras, María Jesús Fernández Aceñero

**Affiliations:** 1 Department of Legal Medicine, Psychiatry and Pathology, School of Medicine, Complutense University of Madrid, 28040 Madrid, Spain; luis.ortega@salud.madrid.org (L.O.M.); jmariajesus.fernandez@salud.madrid.org (M.J.F.A.); 2 Department of Pathology, Hospital Clínico San Carlos, Health Research Institute of the Hospital Clínico San Carlos (IdISSC), 28040 Madrid, Spain; elenamilagrosa.molina@salud.madrid.org; 3 Department of Basic Medical Sciences, School of Medicine, Rey Juan Carlos University, Móstoles, 28933 Madrid, Spain; lourdes.estrada@hospitalreyjuancarlos.es (L.E.M.); soledad.garcia@urjc.es (S.G.G.d.l.H.); 4 Department of Pathology, Rey Juan Carlos Hospital, Móstoles, 28933 Madrid, Spain; 5 Biobank, Hospital Clínico San Carlos, 28040 Madrid, Spain

**Keywords:** age, gastric cancer, clinicopathological features, prognosis, younger

## Abstract

**Simple Summary:**

The impact of age on gastric cancer (GC) features is controversial, and most studies have been conducted in Asian countries. Clarifying this issue can enhance our disease understanding, refine risk stratification models, and aid in the development of personalized therapeutic approaches for GC. This study aimed to evaluate the influence of age at diagnosis on the clinicopathological features, prognosis, and management of a specific cohort of Spanish patients with resected GC. Younger patients showed a higher prevalence of flat, diffuse, high-grade tumors, signet-ring cells, perineural infiltration, D2 lymphadenectomies, and adjuvant therapy. However, we did not detect prognostic differences between age groups. Our results suggest that younger patients may require more aggressive treatment due to adverse clinicopathologic features, but the lack of prognostic differences observed indicates the need for further investigation into the complex interplay between age, clinicopathologic factors, and long-term outcomes in GC.

**Abstract:**

The impact of age on various aspects of gastric cancer (GC) remains controversial. Clarifying this issue can improve our understanding of the disease, refine risk stratification models, and aid in personalized therapeutic approaches. This study aimed to evaluate the influence of age at diagnosis on the clinicopathological features, prognosis, and management of a specific cohort of Spanish patients with resected GC. The study encompassed 315 patients treated at a single tertiary hospital in Spain, divided into two age-based subgroups: ≤65 years and >65 years. The mean and median ages at diagnosis were 72 and 76 years. Most tumors were diagnosed at pT3 stage (49.2%), and 59.6% of patients had lymph node metastases. 21.3% of cases were diagnosed with GC at age ≤ 65 years. Younger patients showed a significantly higher prevalence of flat, diffuse, high-grade tumors, signet-ring cells, perineural infiltration, D2 lymphadenectomies, and adjuvant therapy. They also exhibited a higher rate of recurrences, but had a significantly longer follow-up. Kaplan-Meier curves indicated no significant prognostic differences based on age. Finally, age did not independently predict overall survival or disease-free survival. Our results suggest that younger patients may require more aggressive treatment due to adverse clinicopathologic features, but the lack of prognostic differences among age groups in our cohort indicates the need for further investigation into the complex interplay between age, clinicopathologic factors, and long-term outcomes in GC.

## 1. Introduction

According to the latest GLOBOCAN estimates published in 2020, gastric cancer (GC) ranks as the fifth most common cancer worldwide and the fourth leading cause of cancer-related deaths [1]. In Western countries, the majority of GC patients are diagnosed at advanced stages and prognosis is poor, with an estimated 5-year overall survival (OS) rate of less than 30% [2]. GC is generally considered an age-related disease, with most cases diagnosed in individuals aged 65 years or older [3,4]. However, in recent years, an increasing incidence of GC among younger patients has been observed by several researchers [5,6]. 

Notably, certain Asian countries, where GC incidence is higher, have implemented screening protocols that have led to earlier detection of the disease at younger ages [7]. Asian and Western countries also show differences in the clinical, histological, molecular and prognostic features of GC [8,9,10,11,12]. Most studies investigating the impact of age at diagnosis on the clinicopathological and prognostic features of GC have been conducted in Asian populations [3]. These studies have identified an association between younger age and the presence of diffuse-type and high-grade tumors [13]. However, the relationship between age, other clinicopathologic features, and, more intriguingly, prognosis, remains controversial. 

Given these observations, it is imperative to comprehensively evaluate the influence of age at diagnosis on GC features. Through investigating this relationship, we can enhance our understanding of the disease and uncover the unique factors that contribute to GC development and progression in different age groups. Additionally, we have the potential to refine risk stratification models and develop personalized therapeutic approaches tailored to specific age groups. Such insights may ultimately lead to improved outcomes for GC patients.

The significance of this study is further underscored by the scarcity of evidence regarding Western patients. As far as we know, this analysis represents the first comparative study assessing the impact of age at diagnosis on Spanish GC patients. Our objective is to evaluate how age at diagnosis influences the clinicopathological features, prognosis, and management of a select Spanish population with resected GC.

## 2. Materials and Methods

This retrospective study encompassed all patients who underwent elective surgery with curative intent for GC at a tertiary referral hospital in Madrid, Spain, from 2001 to 2019. The clinical records were thoroughly reviewed, and data on demographics, clinical characteristics, and follow-up information were collected. This included patient age, gender, symptoms, smoking and drinking habits, tumor location, extent of gastrectomy and lymphadenectomy, administration of adjuvant therapy, GC progression, and cause of death. Gross findings, such as tumor size and macroscopic type according to the Borrmann classification, were obtained from the Surgical Pathology Department’s database (PatWin). 

The cases were categorized into two subgroups based on age: ≤65 years and >65 years. Concerning the selection of the cutoff value, reported cutoff points vary among different studies. In Western populations, GC is diagnosed at more advanced ages, and the mean age of our patients was high. Therefore, we opted for the highest cutoff point used in the reviewed studies [14,15,16,17]. Furthermore, this chosen cutoff point aligns with the retirement age in our country, which carries both vital (lifestyle) and economic implications. It also correlates with the cutoff point for the onset of increased GC incidence: the majority of cases occur from the ages of 65–69 onwards, and “older” patients are usually defined as patients over 65–70 years [18].

### 2.1. Inclusion Criteria

A total of 377 patients underwent surgery for GC at our institution between 2001 and 2019. After data collection, patients who received neoadjuvant therapy, had metastatic tumors at the time of diagnosis, or underwent R1 or R2 resections were excluded from the study. The final analysis included 315 patients.

### 2.2. Histopathological Study

Specimens were fixed in formalin, embedded in paraffin, sectioned, and stained with hematoxylin and eosin. All slides were independently reviewed by two pathologists who were blinded to the outcome. A detailed protocol for histologic evaluation was followed, and discordant cases were jointly reviewed. Key microscopic features assessed included tumor type (according to the Laurén and WHO classifications), histologic grade, tumor depth, presence of signet-ring cells, growth pattern, perineural infiltration, lymphovascular invasion, surgical margins, number of dissected lymph nodes (LN), and number of involved LN. Tumors were staged according to the 8th edition of the AJCC-TNM classification [19].

### 2.3. Immunohistochemical Study

Five tissue microarrays (TMA) were constructed for immunohistochemical (IHC) study, which included a subgroup of cases from this cohort (*n* = 180). Each TMA block contained two cores per case, representing the tumor center and leading edge. The TMA were assembled using the MTA-1 tissue arrayer from Beecher Instruments (Sun Prairie, WI, USA). Cores with a diameter of 1 mm were punched from pre-selected tumor regions in paraffin-embedded tissues. 2-μm sections were obtained from the TMA blocks for IHC staining.

To prepare the slides for staining, they were deparaffinized by incubating at 60 °C for 10 min and then incubated with PT-Link from Dako (Agilent, Glostrup, Denmark) for 20 min at 95 °C in a high pH buffered solution. Peroxidase blocking reagent from Dako (Denmark) was used to block the peroxidase activity. The biopsies were incubated with primary antibodies for 20 min, followed by incubation with the appropriate anti-Ig horseradish peroxidase-conjugated polymer (EnVision, Dako, Denmark) to detect the antigen-antibody reaction. The sections were visualized using 3,3′-diaminobenzidine as a chromogen for 5 min and counterstained with hematoxylin.

The TMA block sections were immunostained for HERCEPTEST, p53, E-cadherin, MLH1, PMS2, MSH2, and MSH6 using pre-diluted antibodies from Dako (Denmark), as previously mentioned. Positive and negative controls were included for the IHC reactions. HERCEPTEST was evaluated according to the CAP recommendations [20]. The GC cases were divided into molecular subgroups based on the classification of the Asian Cancer Research Group, as described in a previous study [21,22]. 

### 2.4. Statistical Analysis

All data were stored in an anonymized Excel file and analyzed using the IBM SPSS 20.0 statistical package. The study encompassed all patients who fulfilled the pre-established inclusion criteria; hence, a prior sample size estimation was not conducted. Nevertheless, based on the enrolled patient population, we conducted a post hoc power analysis which indicated our ability to detect differences of at least 8% in the percentages of the outcome measure between the groups, with a statistical power of 80% (beta-error of 0.2) and an alpha error level of 0.05. This analysis resulted in an estimated required sample size of 294 patients. Descriptive statistics are presented as percentages and frequencies for qualitative variables and as means with standard deviation (SD) or medians with ranges for quantitative variables, as appropriate. The normal distribution of quantitative variables was confirmed with the Kolmogorov-Smirnov test. Univariable associations were analyzed using the chi-square test. Student’s *t*-test was used to compare mean values of quantitative variables with two categories, while analysis of variance (ANOVA) was used for variables with more than two categories. A significance level of *p* < 0.05 was established.

OS was defined as the period from GC diagnosis to death, while disease-free survival (DFS) was defined as the duration from GC diagnosis to disease recurrence or death. OS and DFS curves were estimated using the Kaplan-Meier method, and the log-rank test was employed to assess significance. 

Multivariate Cox regression models for OS and DFS were calculated. The backward stepwise method was applied, and models were adjusted for potential confounders. The following covariates were included in the models: patient sex, age at diagnosis, tumor size, histologic type, presence of signet-ring cells, tumor grade, lymphovascular invasion, perineural infiltration, T stage, and N stage.

## 3. Results

### 3.1. Clinicopathological Features of GC Patients

The clinicopathological features of our cases are presented in Table 1. The majority of patients were male (54.6%) with a mean age of 72 years. Most patients presented with symptoms at the time of diagnosis (89.9%). Tumors were predominantly located in the antropyloric region (53.4%), followed by the gastric body (31.9%), fundus (6.2%), and cardia (2.3%). 

A small percentage of patients (6.2%) presented with linitis plastica. According to the Borrmann classification, the distribution of lesions was as follows: polypoid (18.8%), fungoid (36%), ulcerated (33.2%), and flat (12%). Regarding histologic features, the majority of tumors were classified as intestinal (60.3%), followed by diffuse (31.7%), and mixed (8%) GC. Most patients were diagnosed at pT3 stage (49.2%), and 59.6% of them had lymph node metastases. Tumors were primarily treated by total or subtotal gastrectomy (26.9% and 73.1% of cases, respectively) with lymphadenectomy. Adjuvant therapy was administered in 17.8% of cases. During the follow-up period, 36.7% of tumors recurred, and 27.4% of patients died due to GC.

### 3.2. Univariate Analysis

All cases were divided into two categories: patients aged ≤ 65 years and patients aged >65 years. Among our cohort, 21.3% of patients were diagnosed with GC at an age younger than or equal to 65 years.

The results of the univariate analysis are summarized in Table 2, Table 3 and Table 4. 

Significant differences were observed between the two age groups in several clinicopathological variables, including macroscopic type (*p* = 0.001), Lauren type (*p* = 0.002), presence of signet-ring cells (*p* = 0.003), high-grade histology (*p* = 0.024), perineural infiltration (*p* = 0.039), administration of adjuvant therapy (*p* < 0.001), and performance of a D2 lymphadenectomy (*p* = 0.006) (Table 2). Odds ratios for each variable are presented in Table 3.

Interestingly, younger patients demonstrated a significantly higher recurrence rate but had higher mean and median follow-up times compared to their elderly counterparts (Table 4). Finally, age at diagnosis was not related to other important prognostic variables such as the mortality rate, pT, pN, or pTNM stage.

### 3.3. Survival Analysis

**Figure 1:**
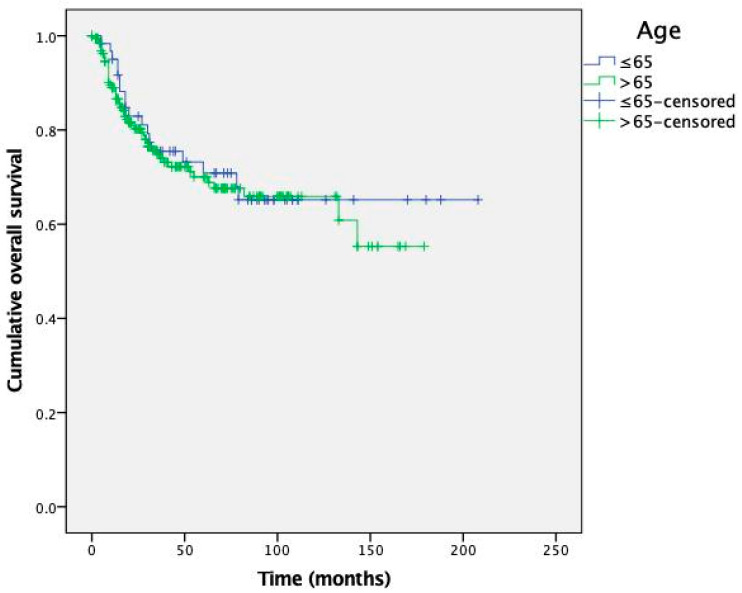
Kaplan-Meier curves for overall survival depending on patient age.

**Figure 2:**
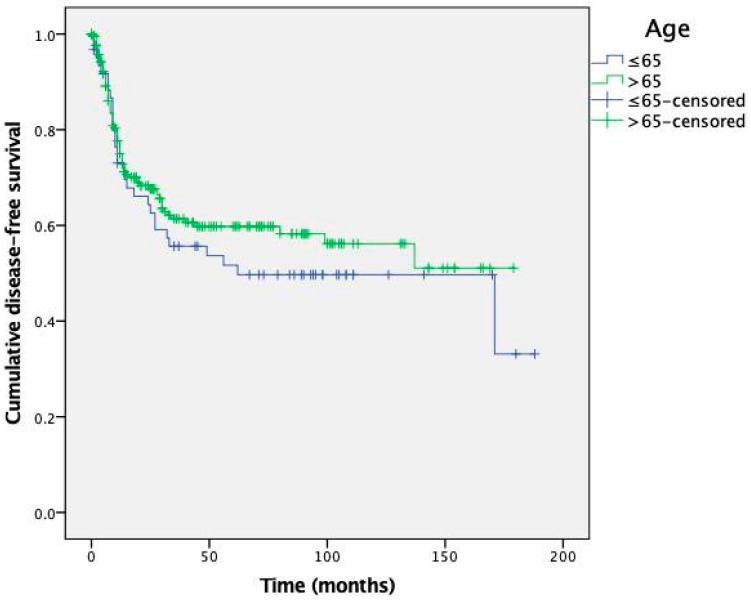
Kaplan-Meier curves for disease-free survival depending on patient age.

Kaplan-Meier curves for OS and DFS are presented in Figure 1 and Figure 2, respectively.

The survival analysis revealed no significant differences between the young and elderly patient groups in terms of both OS and DFS (*p* = 0.696 and *p* = 0.388, respectively). The mean estimated OS (SD) for patients aged ≤65 and >65 years was 147 (11) months and 122 (6) months, respectively. The mean estimated DFS (SD) was 99 (11) and 107 (6) months.

According to the Cox regression analysis, patient age was not an independent prognostic factor for either OS or DFS.

## 4. Discussion

Age is a significant risk factor for cancer development, and most solid tumors are considered to be, at least in part, age-related conditions [3]. In Western countries, GC is typically diagnosed in older individuals. For example, in the United Kingdom the majority of GC cases are diagnosed after the age of 75, with the highest incidence observed in patients aged 85–89 [23]. In our study, the mean and median ages at diagnosis were 72 and 76 years, respectively.

GC has a higher prevalence in Asian countries, with over 60% of new cases being detected in East Asia [24]. Some countries in this region, such as Japan and Korea, have implemented screening protocols. As a result, the age-adjusted incidence rate has shown a downward trend, with patients being diagnosed at earlier stages and younger ages, and exhibiting better survival rates compared to those diagnosed in Western countries [25]. Moreover, geographical disparities between Asian and Western countries have been observed in terms of clinical, histological, and even molecular characteristics [8,9,10,11,12]. It is important to note that the majority of studies investigating the influence of age on GC have been conducted in Asian populations [3]. However, factors such as lifestyle, genetics, and environmental exposures may differ between these regions, potentially influencing the disease progression, treatment response, and overall prognosis of GC. Therefore, it is crucial to investigate the unique characteristics and outcomes of GC in Western populations.

### 4.1. Age at Diagnosis and Clinicopathologic Features of GC

In our analysis, we observed a significant relationship between age at diagnosis and macroscopic type of GC, Laurén classification, presence of signet-ring cells, high histologic grade, and perineural infiltration. Similar associations have been reported by several investigators, with younger patients being more frequently diagnosed with diffuse, signet-ring, and high-grade tumors [13,26,27,28,29]. However, the relationship between patient age and other histologic features has been less extensively studied. A recent study on perineural infiltration in GC found a higher frequency of this feature in patients aged <65 years, consistent with our analysis [14]. Regarding GC macroscopy, authors have variably associated younger patients with ulcerated or flat tumors [30,31]. In our analysis, younger patients exhibited significantly fewer polypoid tumors and a higher proportion of flat tumors compared to their elderly counterparts.

In addition to these features, some authors have identified a relationship between older age at diagnosis and male sex [3,32], higher levels of CA19-9 and CEA [33], and GC located in the upper stomach [34,35]. In our cohort, we did not detect differences in tumor location based on age, as the tumors were evenly distributed in the cardia, fundus, body, and antropyloric region. We found that linitis plastica was more frequently observed in younger patients (11.9% vs. 4.6%), but this difference was not statistically significant (*p* = 0.177). As for patient sex, 52.2% and 55.3% of patients aged >65 and ≤65 years were men, respectively (*p* = 0.657).

Regarding the factors that contribute to the presence of histologically aggressive features in young patients with GC, there is currently a lack of clear understanding. Some researchers propose that this phenomenon might be linked to a higher occurrence of CDH1 mutations in young individuals, which predispose the development of diffuse GC. In this subtype, cells lose their intercellular adhesion properties and display a heightened potential for invasiveness [36]. Diagnostic delays in young patients, resulting from a lower initial suspicion of malignancy, could also play a role in the increased observation of characteristics like lymphovascular invasion, perineural infiltration, and larger tumor size.

In terms of the molecular attributes of GC, older patients tend to exhibit a greater frequency of microsatellite instability (MSI) [37]. On the contrary, younger patients tend to display more mutations in certain genes such as CDH1, RHOA, and CTNNB1 [33]. Interestingly, a recent analysis has identified an enrichment of the CLDN18-ARHGAP fusion gene in GC diagnosed in young adults [38]. Alterations like HER2 amplification, HER2 overexpression, and TP53 mutations seem to occur less frequently in younger patients [39,40]. These observations could be linked to the predominance of diffuse-type GC within this subset of patients. Diffuse tumors exhibit lower rates of MSI and HER2 amplification but with a higher prevalence of CDH1 mutations compared to the intestinal-type GC [41]. Unfortunately, the decreased frequency of actionable molecular alterations in young patients could potentially limit treatment options. This is particularly concerning, given that previous studies have shown a tendency for these patients to be diagnosed at advanced stages. Finally, other investigators have failed to establish substantial distinctions between young and older patients in terms of TP53 mutation rates or HER2 status [42,43].

In our analysis, we did not observe significant differences in the IHC features of GC between older and younger patients. For instance, MSI was observed in 32.2% and 22.7% of patients aged >65 and ≤65 years, respectively (*p* = 0.464). Similarly, we did not observe significant differences in other IHC markers such as HERCEPTEST or p53, or in the IHC classification of GC based on molecular features. This lack of significant findings could be attributed to the absence of notable differences within our studied group, the chosen age threshold, or the relatively infrequent occurrence of detected IHC alterations across both age groups. This is particularly evident in relation to the positive HERCEPTEST results, which were less frequently observed overall. 

### 4.2. Age at Diagnosis: Etiological Hypotheses

Although the causes of GC, many of them dietary and lifestyle-related, and the chronic gastritis—metaplasia—GC process are known and consistent with the appearance of GC in older patients, the underlying causes of GC occurring in young patients, a trend that has been on the rise in recent decades, remain elusive. As suggested by Merchant et al. in their article, multiple hypotheses have been proposed [44]. Firstly, there could be a connection to a higher prevalence of H. pylori infection in young patients, as observed in certain subpopulations [45]. However, the incidence of H. pylori infection has been reported to be declining in recent years, primarily due to economic growth, improvements in food preservation, and effective treatments. Within our cohort, a direct comparison of H. pylori prevalence based on age was hindered by the lack of prior biopsies, breath tests, or other diagnostic analyses in most of our patients, precluding definitive conclusions in this aspect.

Furthermore, the nature of the inflammatory response triggered by H. pylori appears to vary depending on age: pediatric patients have shown a predominantly regulatory response, whereas in adults, there is a predominance of cytokines associated with the Th1 and Th17 responses [46]. While this is a preliminary hypothesis, it is also possible that differences exist in the type of inflammatory response occurring between young adults and older adults. Autoimmunity could also be a significant etiologic factor in young patients [47].

Other authors have linked the rise of GC in young patients to the global increase in obesity over recent decades [48]. However, these “environmental” theories are challenged by the observation that diffuse-type GC is more frequent in young patients. This variant seems to have a weaker association with environmental factors, including H. pylori infection [49]. Unlike intestinal-type GC, which appears to be more influenced by external factors, understanding the etiology of diffuse-type GC is more complex. While it has generally been associated with genetic factors, the specific genetic alterations relevant beyond the context of familial GC are not yet clear. The link between GC in young patients, genetic factors, and the occurrence of diffuse-type cancer appears more consistent than the connection with environmental factors. In fact, several studies have highlighted specific genetic variants, such as single nucleotide polymorphisms, that correlate with an elevated risk of developing GC [50].

Lastly, other researchers have underscored a higher prevalence of Epstein-Barr virus (EBV) in young patients with GC [43]. This discovery holds promise, given that these tumors exhibit PD-L1 overexpression, potentially rendering immunotherapy a viable consideration for this subgroup of young patients.

However, the existing scientific evidence remains insufficient to definitively identify the primary causal factors of GC in young patients. This is primarily due to the fact that a majority of etiological studies have not stratified patients based on age. Future investigations could yield valuable insights by categorizing patients according to both age and the Laurén classification, thereby potentially unraveling the underlying etiology and pathophysiology of this disease.

### 4.3. Age at Diagnosis and Patient Management

In our cohort, younger patients were treated more aggressively, receiving a higher frequency of adjuvant therapy and undergoing more extensive lymphadenectomies than older patients. Younger patients may be more likely to undergo aggressive treatment approaches due to their generally better overall health and ability to tolerate more intensive therapies. They may receive more extensive surgeries, such as radical gastrectomies or lymphadenectomies, along with additional adjuvant therapies like chemotherapy or targeted therapy. This approach aims to maximize the chances of a successful outcome and reduce the risk of recurrence. On the other hand, older patients, especially those with comorbidities or reduced functional status, may be managed with less aggressive interventions. Currently, the most widely utilized treatment guidelines do not explicitly include age as a standalone factor to consider. However, age is indirectly taken into account by considering the overall health status and comorbidities of the patient. Thus, the treatment plan for older patients may involve less extensive surgeries or organ-sparing procedures, considering their potential limitations in tolerating certain treatments. However, the decision-making process should take into account the patient’s overall health, individual preferences, and quality of life considerations. 

Previous studies have found that age may lead oncologists to undertreat otherwise functional patients [51]. The treatment of cancer in older patients is an increasingly common problem due to the increase in life expectancy, and it should involve a multimodal approach. Most clinical trials have been performed in younger and fit patients, resulting in a lack of evidence on GC treatment in older patients [3]. However, if general health and performance status are acceptable, all treatment modalities can be administered to older patients because previous studies have shown supportive results [52]. As Zhang et al. concluded in their study, “functional” age and not “chronological age” should be the criterion for GC screening and treatment [53]. Vallet-Regí et al. published an interesting algorithm for treating older patients that includes mortality risk with cancer, risk of cancer-related complications, and risk of chemotherapy-related toxicity [54].

### 4.4. Age at Diagnosis and GC Prognosis

In our univariate analysis, we observed a higher rate of cancer recurrences among younger patients (49.2% vs. 33.3%, *p* = 0.023). This finding suggests that individuals in the younger age group were more prone to experiencing the return of the disease compared to their older counterparts. However, it is important to consider the potential confounding factors that may have influenced the interpretation of these results. One important factor to take into account is the duration of follow-up. Younger patients generally have a longer life expectancy and may have been monitored for a more extended period. Consequently, there is a higher likelihood of detecting cancer recurrences within that extended follow-up timeframe. This could partly explain the higher occurrence of recurrences observed in the younger age group, as they were followed up for a significantly longer duration compared to older patients (means: 63 vs. 49 months, *p* = 0.001). The median follow-up duration for younger patients was 51 months, whereas for older patients it was 25 months. Furthermore, our analysis revealed no significant relationship between patient age, death due to GC and pT, pN, or TNM stage. 

To further explore the impact of age on GC prognosis, a survival analysis was performed using Kaplan-Meier curves. The analysis revealed no prognostic differences between patients aged ≤ 65 and >65 years. Additionally, age at diagnosis did not independently predict prognosis according to our multivariate analysis. Therefore, the higher occurrence of recurrences in the younger age group may be explained by the longer follow-up period they underwent, due to a lower mortality rate from causes other than GC and a generally higher life expectancy.

Previous studies investigating the impact of age on GC prognosis have yielded contradictory results. Several authors have reported the presence of more aggressive and advanced tumors in younger patients [55,56,57]. A recent systematic review found that younger age is a risk factor for developing peritoneal metastases [58]. However, in our study we did not detect differences in the type of recurrence (distant vs. locoregional) depending on patient age. Additionally, several investigators have observed higher stage tumors and increased mortality rates in younger patients, although no significant differences in survival were found after adjusting for tumor stage [32,59]. Visontai Cormedi et al. found no survival differences among young, older adult, and elderly patients, which is supported by other studies as well [26,56,60]. However, some authors have discovered that younger patients, particularly very young ones, exhibit a significantly poorer prognosis compared to elderly patients [61,62]. Conversely, other authors have found that elderly patients have a significantly worse prognosis than younger patients [63,64]. Guner et al. and Kulig et al. suggested that old age is associated with worse DFS and OS in GC patients, respectively [65,66]. These findings are supported by other authors, such as Talebi et al., who identified age >60 years as an independent prognostic factor for worse prognosis in GC [67]. Another recent study performed in China also identified older age as an independent prognosticator [68]. 

It is important to note that some studies have attributed a worse prognosis to younger or elderly patients based on comparing OS and DFS rates. However, caution should be exercised in interpreting these results, as mean and median OS and DFS can be influenced by the duration of follow-up and deaths unrelated to GC. Kaplan-Meier curves provide a more accurate estimation of survival, as they consider both censored and uncensored cases and estimate the probability of survival across different time periods.

In summary, the relationship between age and prognosis in GC, as observed in previous studies, can be attributed to various factors. Firstly, younger patients tend to exhibit adverse histological characteristics more frequently than their older counterparts, resulting in the emergence of aggressive tumors with a propensity for rapid progression. Secondly, there often exists a lower clinical suspicion of malignancy in younger patients, potentially leading to delays in diagnostic tests and the eventual diagnosis of GC at more advanced disease stages. Thirdly, these patients often undergo more intensive treatment approaches compared to older individuals, which could potentially positively impact their prognosis. Lastly, their generally better overall health and fewer comorbidities can contribute to enhanced treatment tolerance and adherence, thereby reducing the occurrence of undesirable side effects. In our study, the observation that younger patients with resected GC share a comparable prognosis to older patients might be attributed to the likelihood that more intensive treatment regimens have contributed to minimizing the disparities in tumor aggressiveness linked to age.

## 5. Conclusions

In our study, we found that younger patients with resectable GC exhibited distinct clinicopathological characteristics such as flat morphology, diffuse tumors, high histologic grade, presence of signet-ring cells, and perineural infiltration. Furthermore, our findings revealed that these patients received more aggressive treatment, including extensive lymphadenectomies and a higher frequency of adjuvant therapy.

Interestingly, despite the higher rate of recurrences observed in younger patients, we did not observe any significant differences in terms of prognosis when comparing younger and older patients in our cohort. It is worth noting that the higher rate of recurrences in younger patients with resected GC may be attributed to the longer follow-up duration for this group.

The implications of these findings are important for the clinical management of GC, emphasizing the need for personalized treatment approaches that take into account age and associated clinicopathologic features. Our results suggest that younger patients may require more aggressive treatment strategies due to the presence of adverse clinicopathologic features. However, the lack of prognostic differences between age groups in our cohort indicates the need for further investigation into the complex interplay between age, clinicopathologic factors, and long-term outcomes in GC. Additional research is necessary to validate our results and explore additional factors that may influence the prognosis of GC in different age groups.

## 6. Strengths and Limitations of Our Study

### 6.1. Strengths

This study presents a cohesive set of findings derived from a group of Western patients hailing from Spain. All patients were resectable cases diagnosed and treated in a tertiary hospital, and all tumors were independently reviewed by two pathologists following a detailed histological protocol.

### 6.2. Limitations

This study is retrospective in nature, which inherently introduces potential limitations in terms of data collection, inference of causality and susceptibility for biases. GC is less prevalent in Western countries compared to Asian countries, leading to a smaller pool of patients available for analysis. The majority of our participants were of an advanced age at the time of diagnosis (over 65 years), which could potentially diminish the statistical robustness when comparing different age groups. Our cohort represents a homogeneous Spanish population of resectable patients; thus, conclusions about other ethnicities, particularly those originating from Asian countries, and about non-surgical cases should not be extracted.

## Figures and Tables

**Table 1:** Clinicopathological features of the cases included in our study.

Feature		Valid % (*n*)
Age (years) [mean (SD ^a^)]		72 (12)
Age (years) [median (min-max)]		76 (32–93)
>65 years		78.7 (248)
Male		54.6 (171)
Symptomatic	Symptomatic	89.9 (231)
	Local symptoms	65.2 (167)
	Systemic symptoms	53.9 (138)
Size (mm) [mean (SD)]		43 (24)
Location	Cardia/GEJ ^b^	2.3 (7)
	Fundus	6.2 (19)
	Body	31.9 (98)
	Antrum	53.4 (164)
	Linitis plastica	6.2 (19)
Macroscopic type	Polypoid	18.4 (58)
	Flat	12.1 (38)
	Ulcerative	34 (107)
	Fungoid	35.6 (112)
Laurén	Intestinal	60.3 (190)
	Diffuse	31.7 (100)
	Mixed	7.9 (25)
WHO ^c^	Tubular	56.8 (109)
	Discohesive	31.8 (61)
	Mucinous	1.6 (3)
	Mixed	9.9 (19)
Signet-ring cells		32.8 (102)
Histologic grade (high)		50.2 (148)
Necrosis		22.1 (69)
Perineural infiltration		37 (115)
Lymphovascular invasion		37.3 (116)
Growth pattern (infiltrative)		64.3 (151)
Desmoplasia		48.4 (108)
pT	pT1	15.2 (48)
	pT2	20 (63)
	pT3	49.2 (155)
	pT4	15.6 (49)
pN	pN0	40.4 (122)
	pN1	17.5 (53)
	pN2	20.2 (61)
	pN3	21.9 (66)
Lymph node ratio [mean (SD)]		0.2 (0.27)
Metastatic lymph nodes [mean (SD)]		4.1 (6.5)
Lymph nodes retrieved [mean (SD)]		21.9 (14.7)
pTNM stage	I	26 (78)
	II	31.7 (95)
	III	42.3 (127)
Adjuvant therapy		17.8 (48)
Gastrectomy	Total	26.9 (83)
	Subtotal	73.1 (226)
Lymphadenectomy	D1	15.7 (50)
	D2	27.4 (87)
	Not specified	43.1 (137)
Recurrence		36.7 (106)
DFS ^d^ (months) [mean (SD)]		41 (44)
Death		27.4 (72)
Overall survival (months) [mean (SD)]		45 (44)

^a^ SD: standard deviation; ^b^ GEJ: gastroesophageal junction; ^c^ WHO: World Health Organization; ^d^ DFS: disease-free survival.

**Table 2:** Univariate analysis. Clinicopathological variables significantly related to patient age.

Feature		≤65 Years	>65 Years	*p*
Macro ^a^	Polypoid	19.4%	18.1%	0.001
	Flat	25.4%	8.5%	
	Ulcerative	34.3%	33.9%	
	Fungoid	20.9%	39.5%	
Laurén	Intestinal	41.8%	65.3%	0.002
	Diffuse	47.8%	27.4%	
	Mixed	10.4%	7.2%	
Signet-ring cells		47.8%	28.7%	0.003
Histologic grade (high)		63%	46.8%	0.024
Perineural infiltration		47.8%	34%	0.039
Adjuvant therapy		40%	10.8%	<0.001
D2 lymphadenectomy		85.7%	57.8%	0.006

^a^ Macro: macroscopic type.

**Table 3:** Univariate analysis. Odds ratio for the main variables significantly associated with patient age.

Feature	OR, ≤65 Years (95% CI ^a^) *
Laurén (diffuse)	2.4 (1.4–4.2)
Macroscopic type (fungoid)	0.4 (0.2–0.8)
Macroscopic type (flat)	3.7 (1.8–7.5)
Signet-ring cells	2.3 (1.3–4)
Histologic grade (high)	1.9 (1.1–3.4)
Perineural infiltration	1.8 (1.1–3.1)
Adjuvant therapy	5.5 (2.8–11)
D2 lymphadenectomy	4.4 (1.4–13.5)
Recurrence	1.9 (1.1–3.4)

^a^ CI: confidence interval; * Odds ratio has been calculated using the subgroup of patients aged >65 years as a control.

**Table 4:** Univariate analysis. Association between patient age and outcome and stage-related variables.

Feature		≤65 Years	>65 Years	*p*
Recurrence		49.2%	33.3%	0.023
Follow-up (months, mean)		63	49	0.001
Death due to gastric cancer *		*29%*	*26.9%*	*0.738*
*pT*	*pT1*	*14.9%*	*15.3%*	*0.994*
	*pT2*	*19.4%*	*20.2%*	
	*pT3*	*50.7%*	*48.8%*	
	*pT4*	*14.9%*	*15.7%*	
*pN*	*pN0*	*35.4%*	*41.6%*	*0.620*
	*pN1*	*30%*	*16.7%*	
	*pN2*	*17.7%*	*20.8%*	
	*pN3*	*25.8%*	*20.8%*	
*pTNM stage*	*I*	*23.3%*	*26.7%*	*0.788*
	*II*	*35%*	*30.8%*	
	*III*	*41.7%*	*42.5%*	

* italics: variables not significantly associated with patient age (*p* > 0.05).

## Data Availability

Data will be available on request.
